# Model based estimation of the SARS-CoV-2 immunization level in austria and consequences for herd immunity effects

**DOI:** 10.1038/s41598-022-06771-x

**Published:** 2022-02-21

**Authors:** Martin Bicher, Claire Rippinger, Günter Schneckenreither, Nadine Weibrecht, Christoph Urach, Melanie Zechmeister, Dominik Brunmeir, Wolfgang Huf, Niki Popper

**Affiliations:** 1 Institute of Information Systems Engineering, TU Wien, 1040 Vienna, Austria; 2 dwh GmbH, 1070 Vienna, Austria; 3 Association for Decision Support for Health Policy and Planning (DEXHELPP), 1070 Vienna, Austria; 4 Department of Laboratory Medicine, Vienna Healthcare Group, Hietzing Hospital, 1140 Vienna, Austria; 5 Karl Landsteiner Institute for Clinical Risk Management, 1130 Vienna, Austria

**Keywords:** Computational models, Health policy, Viral infection, Epidemiology, Computational science, Applied mathematics

## Abstract

Several systemic factors indicate that worldwide herd immunity against COVID-19 will probably not be achieved in 2021. On the one hand, vaccination programs are limited by availability of doses and on the other hand, the number of people already infected is still too low to have a disease preventing impact and new emerging variants of the virus seem to partially neglect developed antibodies from previous infections. Nevertheless, by February 2021 after one year of observing high numbers of reported COVID-19 cases in most European countries, we might expect that the immunization level should have an impact on the spread of SARS-CoV-2. Here we present an approach for estimating the immunization of the Austrian population and discuss potential consequences on herd immunity effects. To estimate immunization we use a calibrated agent-based simulation model that reproduces the actual COVID-19 pandemic in Austria. From the resulting synthetic individual-based data we can extract the number of immunized persons. We then extrapolate the progression of the epidemic by varying the obtained level of immunization in simulations of an hypothetical uncontrolled epidemic wave indicating potential effects on the effective reproduction number. We compared our theoretical findings with results derived from a classic differential equation SIR-model. As of February 2021, $14.7\%$ of the Austrian population has been affected by a SARS-CoV-2 infection which causes a $9\%$ reduction of the effective reproduction number and a $24.7\%$ reduction of the prevalence peak compared to a fully susceptible population. This estimation is now recomputed on a regular basis to publish model based analysis of immunization level in Austria also including the fast growing effects of vaccination programs. This provides substantial information for decision makers to evaluate the necessity of non pharmaceutical intervention measures based on the estimated impact of natural and vaccinated immunization.

## Introduction

According to the WHO chief scientist Dr. Soumya Swaminathan (interview on 2021.01.11, Deutsche Welle TV), herd immunity with respect to the ongoing COVID-19 pandemic will likely not be reached in 2021. Despite increasing availability and roll out of vaccines and (partial) immunization of individuals/persons with a recent infection, the spread of the pandemic cannot be mitigated without further non-pharmaceutical interventions (NPIs) such as social distancing and increased hygienic awareness. Nevertheless, one can expect that an increasing number of people immunized by having been exposed to the virus will at least slightly slow down the current spreading of SARS-CoV-2. We furthermore want to denote this by the term *herd effect*.

To assess this effect, first, the total number of persons already exposed to the virus needs to be estimated. This number generally consists of the cumulative number of detected SARS-CoV-2 infections and the corresponding amount of cumulative undetected infections that occurred in the background. Since the ratio between these cohorts depends on the reporting system, it is very difficult to transfer findings between different countries. The ratio is most often estimated using randomized studies screening for antibodies. But these studies are very time and cost intensive and therefore rarely performed on a regular basis. Other approaches rely on indirect methods such as estimates for the infection fatality ratio and the case fatality ratio^1^.

Given the number of persons already exposed to the virus, we evaluate the herd effect using modeling and simulation scenarios. Hereby we will compare the disease spread on the example of fictional epidemic waves with and without immunity. We assume that exposed persons developed sufficient antibodies to protect from secondary infections, but also elaborate the limitations and consequences of this assumption. As outcome measures, we investigate the reduction of the effective reproduction number and of the peak prevalence number.

For both tasks, we apply an agent-based model (ABM) in combination with data from performed cross-sectional SARS-CoV-2 prevalence and serological tests in Austria. This ABM has already been used for several scientific studies such as the investigation of tracing measures^2^ or the general dynamics of undetected cases^3^, and is one of three models that participate in the official Austrian COVID forecasting panel by the ministry of health (COVID Prognose Konsortium^4^), which publishes short time forecasts on a weekly basis. For the herd effect, we will also compare the results from the ABM with analytical results from a classic differential equation based SIR-model.

Using this experimental setup, we aim to gain a well founded picture of the total number of known and unknown SARS-CoV-2 cases in Austria until February 2021. We provide estimates on how the corresponding immunization level of the population reduces the maximum epidemiological growth. Based thereon, we formulate the necessary efforts for disease containment.

## Methods

In this section, we present a short introduction to the agent-based model, a specification of the used data and a detailed description of the study setup.

### Agent-based simulation model

**Figure 1:**
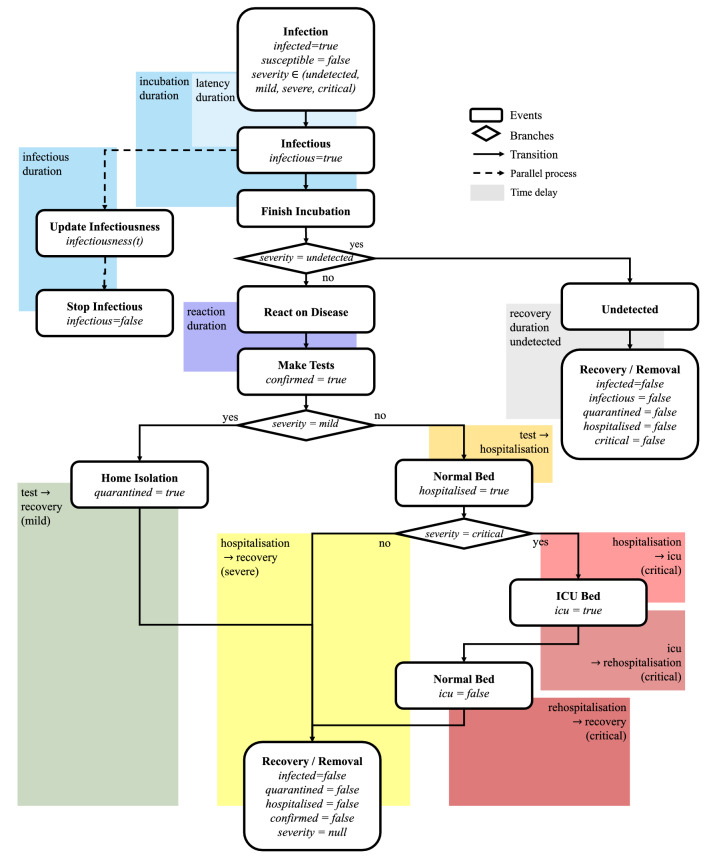
Disease path of an infected agent in the agent-based COVID-19 model. This chart shows an updated version of the one published in the supplemental material of^2^.

We use an agent-based model to simulate the COVID-19 pandemic in Austria. In this model, each agent acts as a statistical representative of a member of the Austrian population, characterized by their age, sex, and place of residence. The number of agents used corresponds to the size of the Austrian population (approx. 9 million people). Using a contact network based on households, workplaces, school and leisure time, the disease can be spread from agent to agent. Once infected, an agent passes through different stages of a SARS-CoV-2 infection, including latency period and incubation period. The model also includes a detection probability which defines whether an agent’s infection will later be confirmed by a positive test result and be recorded in the official vigilance system or whether they remain an undetected case. This parameter is a joint summary of different probabilities such as the chance for an asymptomatic disease progression, the chance to be found in a screening program, and the overall test sensitivity used for detection. A diagram of the full modeled disease pathway is depicted in Fig. 1. By simulating each infection and not only the cases represented in the official vigilance systems, the model can provide a complete picture of the epidemic behavior and the total number of people affected by the virus. A more in-depth description of the simulation model as well as used model parameters are provided in parameter tables in^2^.

As one of the key features of the agent-based model, the model structure allows a very detailed observation of all processes taking place in the simulation. Consequently, the user has almost no limitations regarding definition of the outcome measures. For the present study, our main interest lies in the dynamics, that is time series, of SARS-CoV-2 infected agents—active, cumulative as well as daily new infected. Since we also investigate the detection process of agents, we also observe the detected agents—active, cumulative as well as daily new detected agents—which correspond to the reported case numbers in Austria. Note, that new detected agents correspond to all agents that were detected (received a positive test) on that specific date. Thus, *new detected* differs from *new infected* not only by a factor (detection probability) but also by a time delay.

### Differential equation SIR model

We compare the results from the agent-based model with results from the classic SIR model by Kermack and McKendrick^5^. It is defined by the three coupled differential equations

1 $$\begin{aligned} \dot{S}&=-\alpha I\frac{S}{N}, \end{aligned}$$

2 $$\begin{aligned} \dot{I}&=\alpha I\frac{S}{N}-\beta I, \end{aligned}$$

3 $$\begin{aligned} \dot{R}&=\beta I, \end{aligned}$$

with $N=S+I+R$, and suitable initial conditions. Hereby, the functions *S*, *I* and *R* stand for the time evolution of the total number of susceptible, infectious, and recovered persons in the system.

### Data

In order to parametrize and calibrate the simulation model, a wide range of different data sources has been used. Since these data sources are not specific for the present study, but match the used parameters in previous works, we refer to the parameter tables found in the supplemental material of^2^. With respect to the present study, we want to focus on data sources that reveal information about the detection rate of cases. In Austria, several randomized studies have been performed to estimate the total number of current or past SARS-CoV-2 infections.

In April, SORA Institute for Social Research and Consulting determined that $0.33\%$ ($95\%$ CI $0.12{-}0.75\%$) of the population or approximately 28500 ($95\%$ CI $10,200{-}67,400$) people were infected with SARS-CoV-2 between April 4th 2020 and April $6{\text{th}}$ 2020 (sample size = 1544)^6^. Statistics Austria performed two screening programs between April 22nd 2020 and April $25{\text{th}}$ 2020 and between May $26{\text{th}}$ 2020 and May $30{\text{th}}$ 2020^7^. In the first screening program, 1 of 1432 samples was tested positive resulting in a maximum of $0.15\%$ of the population being currently infected (upper bound of the $95\%$-confidence interval) and in the second screening program, none of the 1279 samples were tested positive.

During the second wave of the COVID-19 pandemic in Austria, Statistics Austria performed once more a randomized screening study^7^ and estimated that $3.1\%$ ($95\%$ CI $2.6{-}3.5\%$) of the people aged 16 or older were currently infected with SARS-CoV-2 between November $12{\text{th}}$ 2020 and November $14{\text{th}}$ 2020 and that $4.7\%$ ($95\%$ CI $3.8{-}5.6\%$) showed antibodies, meaning they had already been infected with SARS-CoV-2 before mid/end of October 2020 (sample size = 2263).

### Estimating the immunization level in February 2021

For the estimation of the immunization level, we ran our simulation model for the time span between February 2020 and February 2021. We identified the parameter for the detection rate based on study data and the method published in^3^. This means, we performed a parameter sweep of the detection probability and determined the number of active cases (detected and undetected) for a given date as a function of the detection probability. We then interpolated the detection probability to match the results of randomized screening studies. As the randomized screening studies did not provide age specific data, we derived age-dependent detection probabilities from the overall detection probability by assuming that the virus is spread equally through all age groups and that the mismatch between the age distribution of the confirmed cases and the Austrian population is caused by different detection probabilities for these age groups. A detailed description of these methods can be found in^3^.

In specific, applying these methods to the results of a prevalence study (SORA^6^) conducted in April 2020, we obtain a detection probability of $13\%$ (CI 95% [$2\%$, $86\%$])^3^. The second study (Statistics Austria^7^), performed during the second wave of COVID-19, indicates an increase of the detection probability to $35\%$ (CI 95% [$24\%, 58\%$]). In our simulation model, we assumed these values to be characteristic for the corresponding epidemic wave. We set the detection probability parameter to a probability of $13\%$ from February 2020 until August 15th 2020 and gradually increased it to $35\%$ until September 1st 2020. This date was chosen because by mid August, Austria implemented a broader testing regime, starting with the free of charge testing of returnees from vacation destinations classified as risk regions and later introduction of excessive nationwide antigen testing for surveillance ^8^.

To guarantee that the detected cases in the model match the officially reported case numbers, we proceeded analogous to^2^ and^3^ and calibrated the infection probability parameter as well as all parameters corresponding to governmental policies for this time period using an iterative bisection method. Hereby, the match of the cumulative detected cases and the officially reported numbers for the positive tests is the calibration target. This process is specified as follows:

1. We specify the scenario as realistic as possible, exploiting all possibilities of the ABM to depict policies set by the government (e.g. quarantine, contact tracing, school closure,...). This way, a time-dependent seasonality-factor $S(t)\in [0,1]$ remains as the sole free parameter of the calibration, which essentially temporarily scales the contact-specific infection probability.
2. We define a vector $\vec {t}$ with $t_k = t_0 + k\Delta t, k\in \{1,2,\dots \}$ of event times. We define that the seasonality factor is constant between any two event times, i.e. $S(t)\equiv S_k$ for $t\in [t_{k-1},t_k)$, and define finding the vector $\vec {S}$ with $S_k, k\in \{1,2,\dots \}$ as the goal of the calibration process. Typically $\Delta t=14[d]$ is used, and $t_0$ corresponds with the start time of the simulation.
3. We run the simulation on the time interval $[t_0,t_0+\xi ]$ and fit the value $S_0$ with a bisection algorithm. Hereby, $\xi =\Delta t+5[d]$ to consider, that changes in the infectiousness of the disease require about 5 days to become visible on the level of the detected cases. The target function of the calibration is the root-mean-squared-error of cumulative confirmed cases.
4. We fix the value found for $S_0$ and continue calibrating with $S_1$. We run the simulation in the interval $[t_0,t_1+\xi ]$ and fit $S_1$ with a bisection method. This step is continued *K*-times until the simulation end-time $t_K+\xi$ of the calibration-step matches the end-time $t_{end}$ of the original simulation scenario.
5. The fitted vector $\vec {S}$ is saved and furthermore used as an input to the simulation. The model is hereby calibrated to retrospective data and can be used for arbitrary scenarios.

Ultimately, in order to obtain an estimate of the immunization level in February 2021, we investigate the final state of our simulation runs. The ratio between the total active and past infected agents and the total number of agents in the model (equivalent to the size of the Austrian population) poses the immunization level and hereby the first major outcome of the model. Note that agents who died (1) from COVID-19, (2) from other causes while being infected, and (3) from other causes after being infected are properly removed by the simulation dynamics and do not contribute to the immunization level.

### Estimating the herd effect

It is our aim to assess how the presumably achieved seroprevalence could affect the future progression of the epidemic. To that end, we investigate a fictional, unconfined outbreak of the disease and compare simulation results with different *initial* immunization levels. That means, we assume no implementation of new NPIs, no change in the contact behavior or any other effects on epidemic progression besides decrease of the susceptible population. We will test for sensitivity on the initial immunization (*x*% of the total population) in both models and provide a discussion of the obtained differences and similarities in the used indicators. Moreover, we observe target indicators such as the time-dependent effective reproduction number and the prevalence of infected and detected cases and study them in comparison with a scenario without initially immunized persons.

The effective reproduction number $\mathcal {R}_{\text {eff}}(t)$ is understood as the average number of infections that are generated by an individual that gets infected at time *t*. Since this number cannot be measured directly in reality, statistical models for inferring the reproduction factor from available data (e.g. reported incidence) must be used. These methods must take into account disease specific parameters, such as the incubation or the infectious period, implying a certain simplified model of disease progression. The effective reproductive number is suitable to differentiate between epidemic progression ($\mathcal {R}_{\text {eff}}(t)>1$) and a stable or declining regime ($\mathcal {R}_{\text {eff}}(t) \le 1$). In this study, we rely on a statistical approach that is available in the software packages EpiEstim^9^. For the configuration of the underlying statistical model (serial interval mean and deviation), we use parameters provided by the Austrian Agency for Health and Food Safety^10^. The same approach is used by authorities and agencies in Austria to provide official estimates of the current reproduction number.

Based thereon, we define the *characteristic reproduction factor* $\widehat{{\mathcal{R}_{{{\text{eff}}}} }}$ as the reproductive factor $\mathcal {R}_{\text {eff}}(\hat{t})$ at time $\hat{t}$ chosen such that (1) the spread of the disease is not yet slowed down by a significant reduction of initially susceptible population and (2) the incidence is high enough to allow for a stable evaluation of the effective reproduction number. Thus, the characteristic reproduction factor is attained in the ‘stable’ phase of an epidemic wave lasting either to the onset of herd immunity effects or until the implementation of countermeasures (i.e. a change in the mixing behavior of the population). Analogously, we assume that a distinct peak/maximum in the observed prevalence is attained at some point in time. We rely on this maximum prevalence number $I_{\text{max}}$ as a second indicator for quantifying the reduction of epidemic spread.

We extract above indicators from two different simulation models that reproduce the fictional wave scenario. The first model is the macroscopic SIR model presented in “Differential equation SIR model” and the second model is the ABM introduced in “Agent-based simulation model”. The former is well suited to generate initial estimates of the investigated target values, since many quantities of the model can be calculated without any numerical simulation. The latter is used to get a more detailed picture of the target measures under consideration of heterogeneous mixing (contact networks).

#### Herd effect estimation using a classic SIR model

Even though there is no analytic solution to these differential equations, the equations still allow to investigate several important epidemiological measures analytically, as long as no containment related policy is applied that changes the values of $\alpha$ and $\beta$ dynamically. For example, $\mathcal {R}_{0}$, the basic reproduction rate, is found as the quotient $\mathcal {R}_{0}=\alpha /\beta$. With *S*(*t*) being the population of the susceptible, the effective reproduction rate calculates to

4 $$\begin{aligned} \mathcal {R}_{\text {eff}}(t)=\mathcal {R}_{0}\frac{S(t)}{N}=\frac{\alpha S(t)}{\beta N}. \end{aligned}$$

Considering *I* as a function of *S* allows to solve the ODE system and calculate the maximum of the infectious curve by

5 $$\begin{aligned} I_{\text {max}}:=\sup _{t\in \mathbb {R}^+}(I(t))=\left\{ \begin{array}{l} I(0)+\left( S(0)-\frac{N\beta }{\alpha }\right) +\frac{N\beta }{\alpha }\left( \ln \left( \frac{N\beta }{\alpha }\right) -\ln (S(0))\right) ,\quad \text {if } S(0)>\frac{N\beta }{\alpha }\\ I(0),\quad \text {otherwise}.\end{array}\right. \end{aligned}$$

A mathematical derivation of this formula is found in the Appendix.

#### Herd effect estimation using an agent-based approach

We apply the agent-based model to run two sets of scenarios corresponding to the initial household setting of the immune agents. In the first set, we distribute the immune individuals fully randomly in the population. In the second set, we reuse the final state of the simulation used for estimation of the seroprevalence level (see “Estimating the immunization level in February 2021”) as the initial state of the new simulation. Hereby, we directly transfer all households with immune agents from the previous simulation and import them into the initial agent population of this simulation. In this way, regional and household clusters from the original simulation are preserved and the spread is more realistic. This approach allows to asses the impact of heterogeneous immunization on our results (compare^11^).

For each scenario, we simulate a hypothetical epidemic wave over the course of six months by randomly introducing 40 infected individuals at the start. This initial number was experimentally determined to produce the most stable results for the stochastic agent-based model.

To calculate the defined outcome measures, we export aggregated daily incidence and prevalence data for the infected and the detected population from our agent-based simulations. The time series of the prevalence allows for direct measurement of the peak height (compare with Eq. (5)). We further apply the statistical estimator for the effective reproduction number that is provided by the *epiEstim* package on our simulated case data. Hereby, we distinguish between the reproduction calculated from the simulated case incidence (${\mathcal{R}}_{{{\text{eff}}}}^{{{\text{tot}}}}$) and the one calculated from the detected cases only (${\mathcal{R}}_{{{\text{eff}}}}^{{{\text{det}}}}$). The latter models to the process performed in the real system, the prior is more accurate, but lacks a real world comparator.

### Ethics

The research in this work is solely based on anonymized and aggregated routine data, most of which is available publicly. No experiments on humans/animals were performed whatsoever. Therefore the study did not require any ethical approval.

## Results

### Immunization level

**Figure 2:**
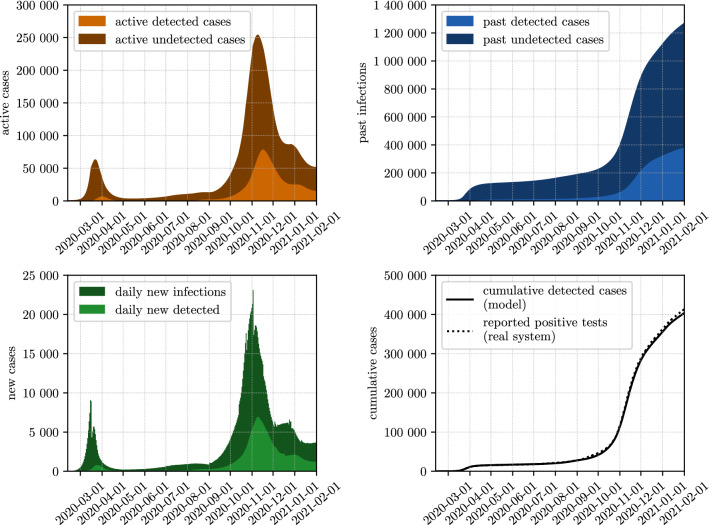
Calibrated model results for the active, past and daily new infections of SARS-CoV-2 from March $12{\text{th}}$ 2020 until January $12{\text{th}}$ 2021. Detected infections denote those people who have received a positive test result and are recorded in the official reporting system. The lower right plot displays a comparison between the calibrated model results and the officially reported case numbers.

**Figure 3:**
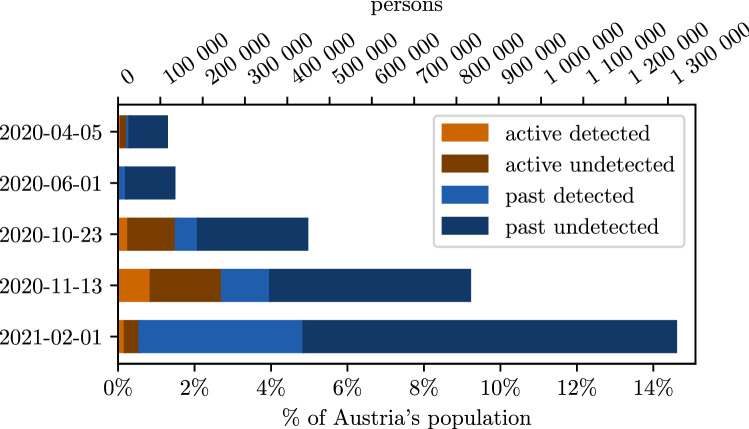
Active and past infections of SARS-CoV-2 for selected dates. Detected infections denote those people who have received a positive test result and are recorded in the official reporting system. The sum of active and past infections denotes the people who have been in contact with SARS-CoV-2 up to that date.

The calibration process of the ABM achieved to match the cumulative detected cases in the simulation with the officially reported case numbers with a root mean square error of $RMSE=3721$ cases. The quality of the fit can be seen in the lower right part of Fig. 2. The other parts of the figure give insights into the dynamics of detected and undetected cases as resulting from the model. Figure 3 shows the share of the population with an active or past infection for selected dates. The selected dates represent the dates relevant for the screening studies (April 5th 2020, mid/end October 2020, November 13th 2020), a date at the end of the first epidemic wave (June 1st 2020) and a current date (February 1st 2021). As of February 1st 2021, $14.1\%$ ($95\%$ CI $8.5{-}36.1\%$) of the Austrian population is already immune to SARS-CoV-2 and $14.7\%$ ($95\%$ CI $9.1{-}36.8\%$) or approximately 1.3 million people ($95\%$ CI 819,000–3.32 million) have been in contact with SARS-CoV-2 due to an active or past infection. From February 2021 also vaccinated persons will be included in this estimation according to the actual assumptions on immunity and reduction of transmission effects. The corresponding data is available on http://www.dexhelpp.at/en/immunization_level. This estimation is recomputed on a regular basis to publish model based analysis of immunization level in Austria also including the fast growing effects of vaccination programs in Austria.

### Quantifying the Herd effect

#### Classic SIR model

Assuming that a certain percentage *x* of the population is initially immune (i.e. $R(0) = \frac{x}{100} N$), the complementary compartment $S(0) + I(0) = N - R(0)$ is reduced by the same amount. Considering that the initial prevalence *I*(0) is negligible small, $S(0)\approx (1-\frac{x}{100}) N$ can be derived.

In the classic SIR model, $\mathcal {R}_{\text {eff}}(0) = \alpha S(0)/\beta N = \alpha (1-\frac{x}{100})/\beta$. We see that the initial value of $\mathcal {R}_{\text {eff}}$ depends linearly on the immunity level of the population and $14.1\%$ initial immune persons result in $\mathcal {R}_{\text {eff}}(0)$ being $85.9\%$ of its original value. Note that this statement does not hold for $\mathcal {R}_{\text {eff}}(t)$ in general, since the time dynamics of the SIR model are nonlinear.

Moreover, we apply Eq. (5) to estimate the impact on the peak of the disease wave. For $I(0)\approx 0$, i.e. $(1-x)S(0) \approx (1-x)N$, we find that

6 $$\begin{aligned} \begin{aligned} I_{\text {max}}(x)&= I(0)+\left( (1-x)S(0)-\frac{N\beta }{\alpha }\right) +\frac{N\beta }{\alpha }\left( \ln \left( \frac{N\beta }{\alpha }\right) -\ln ((1-x)S(0))\right) \\&\approx N\left( \left( (1-x)-\frac{\beta }{\alpha }\right) +\frac{\beta }{\alpha }\ln \left( \frac{\beta }{(1-x)\alpha }\right) \right) \\&=N\left( \left( (1-x)-\frac{1}{\mathcal {R}_{0}}\right) +\frac{1}{\mathcal {R}_{0}}\ln \left( \frac{1}{(1-x)\mathcal {R}_{0}}\right) \right) \end{aligned} \end{aligned}$$

describes the maximum peak height of the epidemic wave, with initially *xN* immune persons. If we investigate the relative reduction of the immunization level $\frac{I_{\text {max}}(0)-I_{\text {max}}(x)}{I_{\text {max}}(0)}$, the total population *N* cancels out of the equation. Applying the formula with typical estimate $\mathcal {R}_{0}=3$, an immunization level *x* of $7.05\%$ results in a reduced peak by $15.4\%$. Moreover, $14.1\%$ provide a $30.1\%$ reduced peak, $21.15\%$ lead to a $44.0\%$ reduced peak and $28.2\%$ initial immune reduce the maximal peak height by $57.1\%$. $\mathcal {R}_{0}=4$ leads to a relative reduction of $13.8\%$ for an immunization level of $7.05\%$, $25.5\%$ for an immunization level of $14.1\%$, $37.7\%$ for an immunization level of $21.15\%$ and $49.4\%$ for an immunization level of $28.2\%$ (see Fig. 7). Easily seen, the relation is almost linear for values *x* close to zero.

Summarizing, linear models can be fitted and used for local approximation of the impact of the number of initially immunized persons on $R_{eff}$ and the maximum peak height. We used these to quantify confidence intervals for the results of the agent-based model.

#### Agent-based model

**Figure 4:**
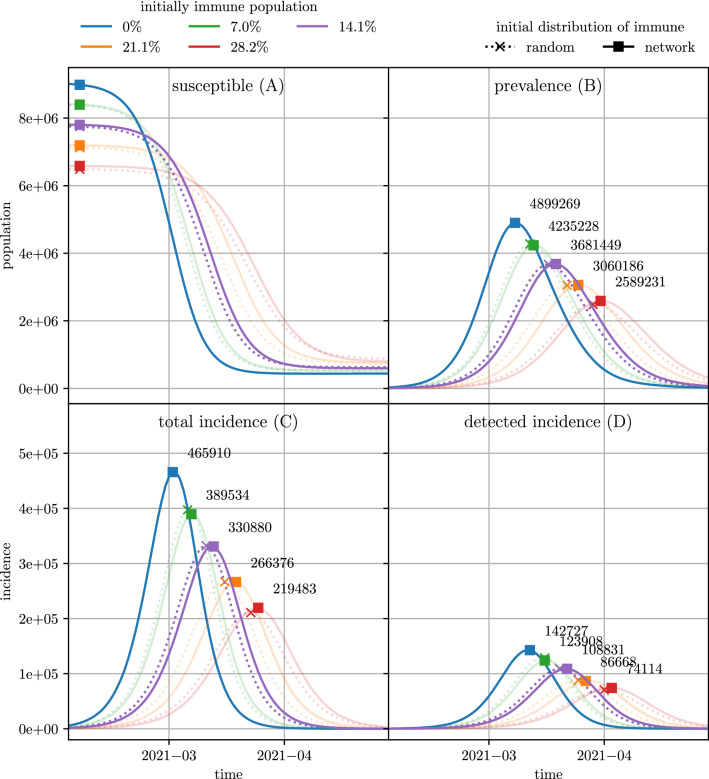
Various outcome measures for scenarios with different initially immunization levels. Simulation results for all scenarios with varying immunization level and distribution of initially immune individuals are depicted. Panel A depicts the number of susceptible individuals and Panel B depicts the number of currently infected individuals. Panel C depicts the daily incidences for all cases of SARS-COV-2 infections and Panel D depicts the daily incidences for the confirmed SARS-COV-2 infections. For each timeline, the maximum of the curve is marked.

Using the agent-based model, we simulated 10 different scenarios of an epidemic wave of SARS-CoV-2 infections with different initial network structures and 5 levels of seroprevalence. Figure 4 depicts various outcome measures of the different scenarios, such as timelines of prevalence and incidence for all cases as well as the detected cases. It can be seen, that a higher immunization level does not only reduce the peak of maximal prevalence but also delays it by 4 days (for $7\%$ immune) to 20–23 days (for $28.2\%$ immune). Furthermore, for all scenarios, the timeline of the new detected cases is delayed by about 9 days compared to the timeline of new infections. This delay occurs because cases are detected about 9 days after their infection as the mean incubation time is 5.1 days and the mean reaction time between symptom onset and positive test results is 3.8 days^3^. The detected cases are also reduced to $35\%$ of all cases which corresponds to the detection probability applied for these scenarios. The network structure of the initially immune individuals has only a small effect on the epidemic curve. Incidences are delayed by 1–2 days with the delay growing with the immunization level but the overall dynamic and the height of the incidence peak is not affected otherwise. Therefore, in the following, we will only present the results for the scenarios with randomly distributed initially immune individuals. The results for the other scenarios can be found in the Appendix.

**Figure 5:**
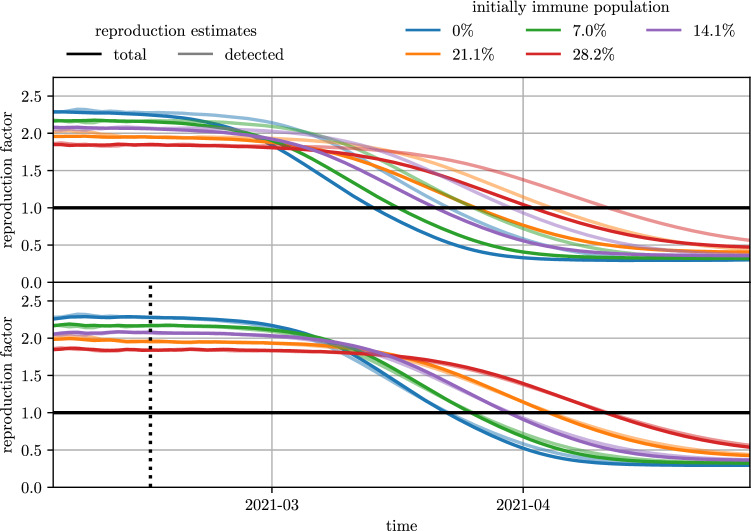
Estimates of the effective reproduction number for different initial immunization level. The effective reproduction number has been evaluated both on the total new cases as well as on the detected new cases. In the lower plot, the evaluation based on all new cases has been shifted to account for the time delay between infection and detection date. The black dotted line indicates the date (2021-02-14) used for evaluation of $\hat{\mathcal {R}}_{\text {eff}}^{\text {tot}}$ and $\hat{\mathcal {R}}_{\text {eff}}^{\text {det}}$.

Figure 5 depicts the temporal dynamic of the effective reproduction number in the different scenarios. In the lower part, $\mathcal {R}_{\text {eff}}^{\text {tot}}$ has already been shifted by 9 days to account for the delay between infection and detection date. After this temporal shift, $\mathcal {R}_{\text {eff}}^{\text {tot}}$ and $\mathcal {R}_{\text {eff}}^{\text {det}}$ can be considered as equivalent^9^. Especially in the early phase of the epidemic wave, where the disease can spread uninhibited, the two measures correspond very well and both variants of $\mathcal {R}_{\text {eff}}$ reach the threshold value of 1 at the same point in time.

**Figure 6:**
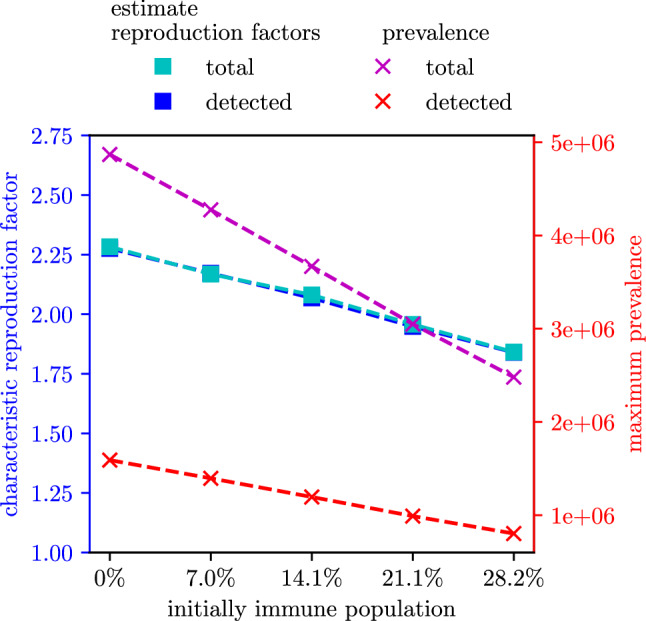
Effective reproduction number $\widehat{{\mathcal{R}}}_{{{\text{eff}}}}$ and maximal prevalence as a function of initial immunization level for the results of the agent-based simulation.

**Figure 7:**
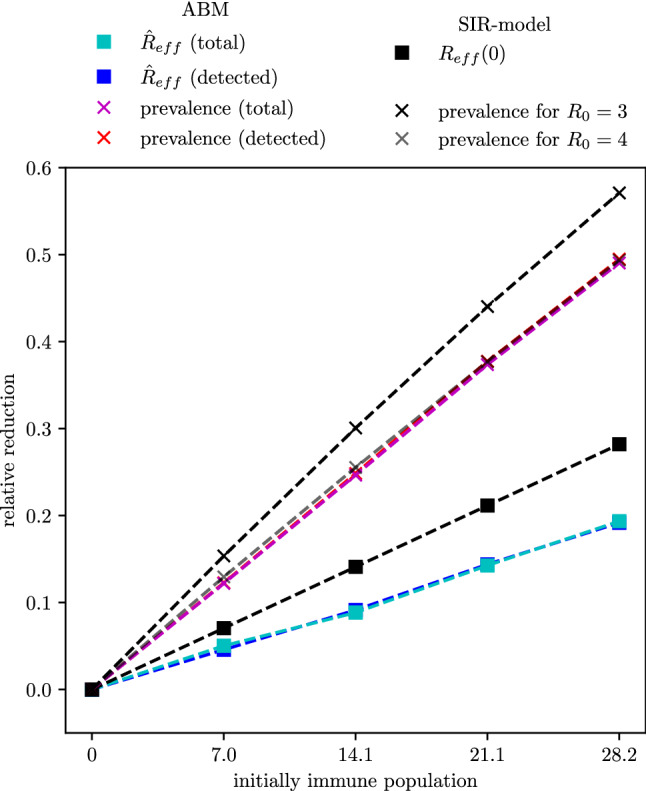
Relative reduction of the effective reproduction number and the peak of the prevalence compared to a fully susceptible population for the results of the classic SIR-model and of the agent-based simulation model.

The values of $\hat{\mathcal {R}}_{\text {eff}}^{\text {tot}}$ and $\hat{\mathcal {R}}_{\text {eff}}^{\text {det}}$, as well as the maximal prevalence, are depicted in Fig. 6. Moreover, Fig. 7 shows the relative reduction of these outcome measures compared to the scenarios with an initially fully susceptible population. For the results of the agent-based simulation model, the relative reduction of the outcome measures is independent on whether the outcome measures were evaluated based on all infections or only on the detected cases. The reduction of $\hat{\mathcal {R}}_{\text {eff}}^{\text {tot}}$ and $\hat{\mathcal {R}}_{\text {eff}}^{\text {det}}$ caused by a higher level of initial immunization follows the linear trend as in the classic SIR-model. However, in the agent-based model, the relative reduction caused by an initial immunization level of $14.1\%$ lies only between $8.8\%$ (for detected cases) and $9.1\%$ (for all cases). The relative reduction of the maximal prevalence matches well with the estimates generated with the classic SIR-model and $\mathcal {R}_{0}= 4$.

## Discussion

An agent-based model was used to give a detailed estimate on the level of immunization among the Austrian population in February 2021 and how this influences the future spread of the pandemic. The modeling and simulation study reveals that with February $1{\text{st}}$ 2021, a total number of 1.328 million Austrian inhabitants have already been exposed to the virus of which 1.319 million are still alive to this date, 7778 died due to COVID-19, and 1030 died after having recovered from the disease. Assuming that the persons being exposed to the virus developed sufficient antibodies for immunization, $14.1\%$ ($95\%$ CI $8.6{-}36.1\%$) of the Austrian population can be considered immune by February 2021. The results of the modeling study indicate that this level of immunization leads to a maximum reduction of $\mathcal {R}_{\text {eff}}$ by 9.1% ($95\%$ CI $5.7\% - 24.6\%$) compared to a fully susceptible population and a reduction of the maximum prevalence by 24.7% ($97.5\%$ CI $15.0{-}63.3\%$, see Fig. 7). Assuming that the virus remains as infectious as it has been in spring 2020, this percentage directly maps to the policies required for containment of the disease.

With respect to quantifying the herd effect, we defined different outcome measures giving similar, yet slightly different pictures. This underlines, that defining consistent outcome measures for assessment of epidemic spread related measures or effects is still a challenging task. In specific, our study shows that even though estimations of the relative reduction of the effective reproduction number due to an initial immunization level can be drawn from the classical SIR-model, these estimations do not directly relate to the results of more complex simulation models. First, the classical SIR-model neglects or oversimplifies some important factors of the modeling of SARS-CoV-2, the most important being the incubation and latency period, the isolation of symptomatic cases and the general stochasticity of the system. While the “naive” SIR-model can be used to determine the theoretical effect a certain immunization level of the population has on $\mathcal {R}_{\text {eff}}$, several extensions to the model are necessary to accurately depict the spread of SARS-COV-2 using differential equations (e.g.^12^). Second, measuring $\mathcal {R}_{\text {eff}}$ in a post-processing step from case numbers gives different results than directly measuring $\mathcal {R}_{\text {eff}}$ from the model parameters since additional parameter assumptions need to be made.

Apart from these differences, comparisons between the results of the agent-based model and the analytic calculations from the classical SIR model show interesting similarities and insights. First of all, the agent-based model results follow the linearity predicted by the analytic calculations: the maximum value of $\mathcal {R}_{\text {eff}}(t)$, approximated by the characteristic reproduction rate, and the maximum peak height of the active cases $I_{\text{max}}$ relate indirect proportionally to the base immunity level of the population. Secondly, the agent-based model also provides a much deeper insight into the disease spread related processes than the classic SIR model. For example, we find different sources for time-delays in different outcomes such as the delay of about 9 days between $\mathcal {R}_{\text {eff}}^{\text {det}}(t)$ and $\mathcal {R}_{\text {eff}}^{\text {tot}}(t)$ (see Fig. 5) and the delay of about 1–2 days caused by a different initial network structure (see Fig. 4). The prior nicely depicts why any kind of COVID-19 countermeasure can only show effects in the reported case numbers at the earliest one week after introduction of the policy and is well known among policy makers. The latter is a less obvious observation and requires deeper analysis. It shows that the disease requires about 1 to 2 days to “break out” of naturally grown immunized networks while it can already spread with higher velocity if the initial network of immunized agents is randomly created. This can be emphasised as a surprising finding of this study, since we would have expected a higher impact of the initial network structure.

Our method to evaluate the total number of people who have been in contact with SARS-CoV-2 differs from other approaches using estimates of the infection fatality rate^1^ and resulting in an estimated immunization level of $7\%$ for Austria in February 2021. Moreover, our method to quantify the effect of this immunization level is vastly different to methods applied for other infectious diseases that rely on observational studies performed before and after vaccination of a large portion of the population^13^^14^.

The results of this study are limited by general limitations of modeling and simulation based research including modeling simplifications and data quality. Like all models on the total amount of infected persons also this work relies on international and national studies and analysis of not detected cases based on measured data. In addition we assumed that a past infection of SARS-COV-2 will always result in immunity long enough for the scope of this study (no loss of immunity). Studies have shown that antibodies against SARS-COV-2 can be found 6 months after an infection in $84\%$ of the cases^15^ and that a previous infection lowers the risk of a new infection by $83\%$^16^. However, more and more reports of reinfections with SARS-COV-2 emerge with the second wave of COVID-19 since fall 2020, indicating that this model assumption might be too general. Yet, since there is still too little data available, we found the evidence not convincing enough to change fundamental parts of the model at the moment. Furthermore, we did not account for the rising number of mutations of SARS-COV-2. As with general reinfections, we did not consider subsequent infections with different mutations. Similarly, we did not vary the overall infectivity of SARS-COV-2. This means, we did not account for potentially higher infectivity of new mutants that slowly replace current strains. Besides complex effects in post infection immunity, our study does also not consider general intervention measures such as restriction of social contacts and vaccination. Hence, we observe theoretical scenarios of an uncontrolled epidemic wave and analyze the isolated impact of natural immunization on the effective reproduction number.

The data sources used for this study also have several limitations. Some of the randomized prevalence studies have been performed at a time with low case numbers (end of April 2020 and May 2020). Here, the sample size of 1432 and 1279, respectively, were not big enough to produce a significant outcome useful for further analyses. Moreover, all prevalence studies only included people age 16 or older and did therefore provide no further insight in the number of undetected cases among children. Finally, the official data used for calibrating the model comes with a great number of reporting bias. Reported cases vary highly with weekends and national holidays or the testing regime applied in the different Austrian federal states.

Next steps include constant reevaluation of the immunization level and its impact on the spread of COVID-19 in Austria, especially focused on the effect of the vaccination program in Austria. This provides substantial information for decision makers to evaluate the necessity of NPI-measures based on the estimated impact of natural and vaccinated immunization at a given moment. Further research will include additional analysis and adaption of modelling parameters once new evidence from randomized screening studies and more reliable data on the risk of reinfection with new virus strains or the role of waning antibodies is available.

## Figures and Tables

**Figure 8:**
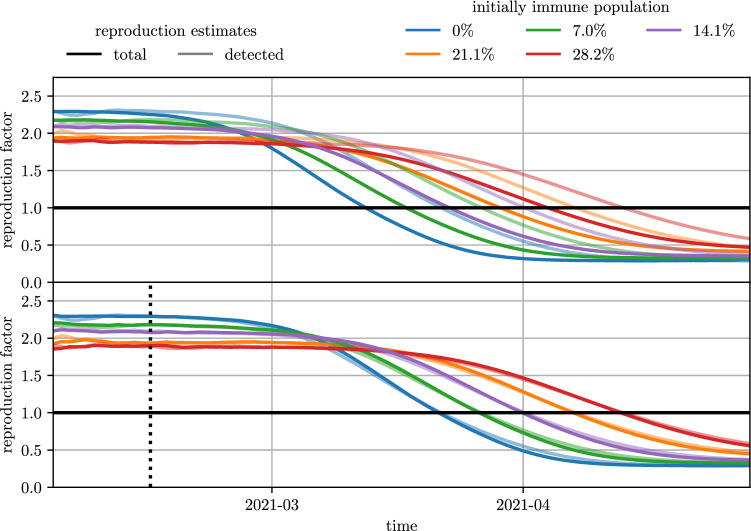
Estimates of the effective reproduction number for different initial immunization level. The effective reproduction number has been evaluated both on the total new cases as well as on the detected new cases. In the lower plot, the evaluation based on all new cases has been shifted to account for the time delay between infection and detection date. The black dotted line indicates the date (2021-02-14) used for evaluation of $\hat{\mathcal {R}}_{\text {eff}}^{\text {tot}}$ and $\hat{\mathcal {R}}_{\text {eff}}^{\text {det}}$.

**Figure 9:**
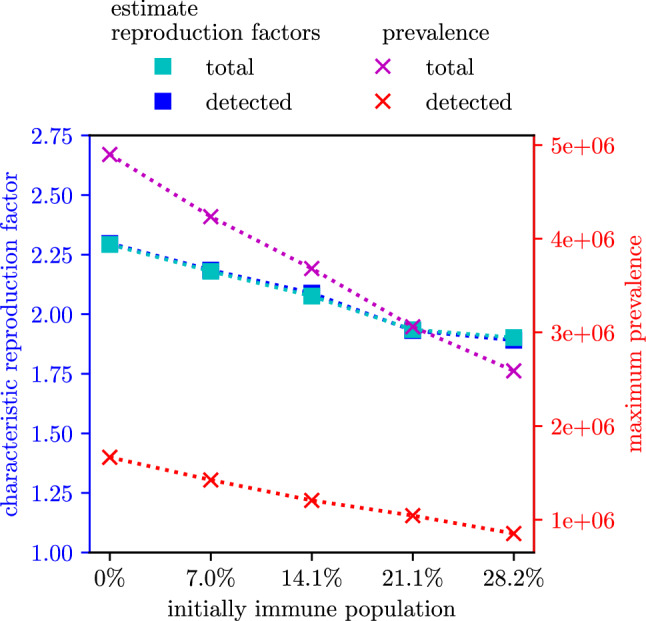
Effective reproduction number and maximal prevalence as a function of initial immunization level for the results of the agent-based simulation.

**Figure 10:**
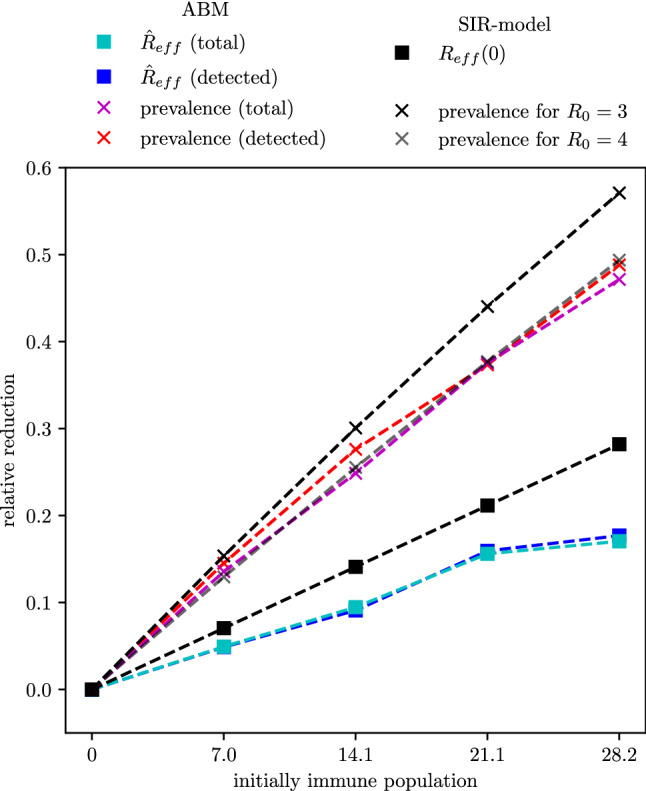
Relative reduction of the effective reproduction number and the peak of the prevalence compared to a fully susceptible population for the results of the classic SIR-model and of the agent-based simulation model.

## A: Derivation of formula (5)

We consider the classic SIR model (1)–(3) with $\mathcal {R}_{0}=\alpha /\beta >1$, initial conditions $I(0),S(0),R(0)>0$, and $I(0)+S(0)+R(0)=N$.

For the first case, we will investigate $S(0)>N\beta /\alpha$. This ensures, that $I'(0)>0$ and the results of the model will show a classic epidemic outbreak.

Since Eq. (1) has a strictly negative right hand side, the resulting function *S* must be monotonically decreasing. Consequently, $S^{-1}$ is a well defined function on the value set of *S* and we may investigate *F* defined by

$$\begin{aligned} F:\text {image}(S(t))\rightarrow \mathbb {R}: u\mapsto F(u)= I(S^{-1}(u)). \end{aligned}$$

With $u=S(t)$ the function results in $F(S^{^-1}(S(t)))=I(t)$ and therefore describes the number of the infectious *I* as a function of the susceptible *S*.

Using the chain rule, and the rule for the differentiation of inverse functions, we derive

$$\begin{aligned} \frac{dF}{du}(u)=\frac{dI}{dt}(S^{^-1}(u))\frac{dS^{-1}}{du}(u)=\frac{dI}{dt}(S^{-1}(u))\frac{1}{\frac{dS}{dt}(S^{-1}(u))}. \end{aligned}$$

Applying the classic SIR Eqs. (1–3) we follow

$$\begin{aligned} \frac{dF}{du}(u)&=\frac{\frac{dI}{dt}(S^{-1}(u))}{\frac{dS}{dt}(S^{-1}(u))}=\frac{\frac{dI}{dt}(t)}{\frac{dS}{dt}(t)}=\frac{\alpha I(S^{-1}(u))\frac{S(S^{-1}(u))}{N}-\beta I(S^{-1}(u))}{-\alpha I(S^{-1}(u))\frac{S(S^{-1}(u))}{N}}\\&=-1+\frac{N\beta }{\alpha }\frac{1}{S(S^{-1}(u))}=-1+\frac{N\beta }{\alpha }\frac{1}{u}. \end{aligned}$$

Since

$$\begin{aligned} 0=\frac{dF}{du}(u)=-1+\frac{N\alpha }{\beta }\frac{1}{S(t)}\Rightarrow u_{\text{max}}:=u=\frac{N\beta }{\alpha } \end{aligned}$$

and

$$\begin{aligned} \frac{dF^2}{du^2}\left( \frac{N\beta }{\alpha }\right) <0, \end{aligned}$$

the value $u_{max}=\frac{N\beta }{\alpha }$ maximizes *F*, as long as it lies in the image of *S*(*t*). This is necessarily true, since $S(0)>u_{\text{max}}$ and $I'(t)>0$ for all $S(t)>u_{\text{max}}$. Therefore, *S* needs to drop below $u_{\text{max}}$ in the course of the epidemic wave and there exists a $t_{\text{max}}>0$ s.t.

$$\begin{aligned} S_{\text{max}}:=S(t_{\text{max}})=u_{\text{max}}. \end{aligned}$$

Finally, we may find the corresponding maximum peak value $I_{\text{max}}=I(t_{\text{max}})=F(S(t_{\text{max}}))$ via integration:

$$\begin{aligned} F(S(t_{\text{max}} )) - F(S(0)) & = F(S_{\text{max}} ) - I(0) = \int_{{S(0)}}^{{S_{\text{max}} }} - 1 + \frac{{N\beta }}{\alpha }\frac{1}{u}du \\ & = \left| { - u + \frac{{N\beta }}{\alpha }\ln (u)} \right|_{{S(0)}}^{{S_{\text{max}} }} = (S(0) - S_{\text{max}} ) + \frac{{N\beta }}{\alpha }(\ln (S_{\text{max}} ) - \ln (S(0))). \\ \end{aligned}$$

Therefore

$$\begin{aligned} I_{\text {max}}= F(S(t_{\text{max}})) = I(0)+\left( S(0)-\frac{N\beta }{\alpha }\right) +\frac{N\beta }{\alpha }\left( \ln \left( \frac{N\beta }{\alpha }\right) -\ln (S(0))\right) , \end{aligned}$$

for the first case with $S(0)>\frac{N\beta }{\alpha }$.

For the second case with $S(0)\le \frac{N\beta }{\alpha }$, we find $I'(t)<0$ for all $t>0$ leading to a monotonically decreasing function *I*(*t*). Consequently, *I* has its maximum at $t=0$.

## B: Results for scenario with network

Figures 8, 9 and 10 display the analogous curves as shown in Figs. 5, 6 and 7 for the scenarios with a model-grown initial immunization network.
